# Clinical Ethics in Gabon: The Spectrum of Clinical Ethical Issues Based on Findings from In-Depth Interviews at Three Public Hospitals

**DOI:** 10.1371/journal.pone.0132374

**Published:** 2015-07-10

**Authors:** Daniel Sippel, Georg Marckmann, Etienne Ndzie Atangana, Daniel Strech

**Affiliations:** 1 Department of Neurology, Carl Gustav Carus University Hospital, Technische Universität Dresden, Dresden, Germany; 2 Center for Regenerative Therapies Dresden (CRTD), Technische Universität Dresden, Dresden, Germany; 3 Institute of Ethics, History and Theory of Medicine, Ludwig-Maximilians-Universität München, Munich, Germany; 4 Department of Neurosurgery, University Medical Center Göttingen, Göttingen, Germany; 5 Institute for History, Ethics and Philosophy of Medicine, Centre for Ethics and Law in the Life Sciences (CELLS), Hannover Medical School, Hannover, Germany; Canadian Agency for Drugs and Technologies in Health, CANADA

## Abstract

**Introduction:**

Unlike issues in biomedical research ethics, ethical challenges arising in daily clinical care in Sub-Saharan African countries have not yet been studied in a systematic manner. However this has to be seen as a distinct entity as we argue in this paper. Our aim was to give an overview of the spectrum of clinical ethical issues and to understand what influences clinical ethics in the Sub-Saharan country of Gabon.

**Materials and Methods:**

In-depth interviews with 18 health care professionals were conducted at three hospital sites in Gabon. Interview transcripts were analyzed using a grounded theory approach (open and axial coding), giving a qualitative spectrum of categories for clinical ethical issues. Validity was checked at a meeting with study participants and other health care experts in Gabon after analysis of the data.

**Results:**

Twelve main categories (with 28 further-specified subcategories) for clinical ethical issues were identified and grouped under three core categories: A) micro level: “confidentiality and information”, “interpersonal, relational and behavioral issues”, “psychological strain of individuals”, and “scarce resources”; B) meso level: “structural issues of medical institutions”, “issues with private clinics”, “challenges connected to the family”, and “issues of education, training and competence”; and C) macro level: “influence of society, culture, religion and superstition”, “applicability of western medicine”, “structural issues on the political level”, and “legal issues”.

**Discussion:**

Interviewees reported a broad spectrum of clinical ethical issues that go beyond challenges related to scarce financial and human resources. Specific socio-cultural, historical and educational backgrounds also played an important role. In fact these influences are central to an understanding of clinical ethics in the studied local context. Further research in the region is necessary to put our study into perspective. As many participants reported a lack of awareness of ethical issues amongst other health care professionals in daily clinical practice, we suggest that international organizations and national medical schools should consider infrastructure and tools to improve context-sensitive capacity building in clinical ethics for Sub-Saharan African countries like Gabon.

## Introduction

For a long time, conceptual and empirical bioethics projects played only a marginal role in Sub-Saharan Africa. As more clinical trials of HIV/AIDS drugs, malaria treatments and vaccines started to be conducted in Africa, scholars in bioethics and health policy analysis began to study the ethical implications of these clinical research projects, including informed consent issues, post-trial access to trial drugs, and setting-relative risk-benefit analysis [1–4]. Capacity building and infrastructure for research ethics and research oversight has been improved in African countries and supported by various stakeholders. However, clinical ethical issues arising in the doctor-patient relationship and in related decision-making areas have received remarkably little attention and have not been studied in a systematic and comprehensive manner. We argue that clinical ethics of everyday practice in the hospital setting has to be seen as a distinct entity compared to research bioethics because of observed and reported differences we cover below. An exploratory PubMed literature search with the search string “ethics” [MeSH] AND “Africa” [MeSH] revealed a variety of “ethical issues”. However, most of the papers we found concerned research ethics in Africa. Only a few papers looked at clinical ethical issues in Africa. The few conceptual papers on clinical ethics that we found were almost all from South Africa, and dealt with a variety of specific challenges, e.g. disclosure of information, informed consent, HIV-testing, abortion, traditional medicine, palliative care, occupational health, organizational ethics, and just allocation of scarce resources at the physician/hospital level, but didn’t give an overview of clinical ethical issues in the clinical context [5–28].

In 2002, UNESCO made ethics a principal priority and in 2008 it became one of five overarching objectives in the “UNESCO Medium Term Strategy 2008–2013” [29,30]. In 2005, the “Universal Declaration on Bioethics and Human Rights” was unanimously adopted by the UNESCO member states. Furthermore the “Global Ethics Observatory” [31], a set of free databases “…intended to become a crucial platform for supporting and advancing ethics activities by assisting Member States and other interested parties to identify experts, establish ethics committees, construct informed policies in the area of ethics, and design ethics teaching curricula” [32] was established by UNESCO. However, just as in bioethical research, the principal focus was on capacity building of national ethics committees for research oversight and health care reforms [33,34] and not on clinical ethics in the everyday hospital setting.

The aim of this study was to analyze the spectrum of clinical ethical issues in various health care settings in a Sub-Saharan country (Gabon) and determine the factors leading to and influencing the issues that were reported. We therefore chose a qualitative research approach, as this is the best mean to get insights into the topic of clinical ethics in Gabon. Qualitative research in the form of openly conducted in-depths interviews allows for a more comprehensive insight into the spectrum of ethical issues that health care professionals in Gabon face in their daily work compared to a questionnaire or other quantitative approaches that could aim at, for example, the investigation of how often these issues occur [35]. For the analysis of the interviews we framed the classification of an “issue” as an “ethical issue” in a rather broad sense as we did not want to exclude any issue that one would not instantly classify as “ethical issue” when coming from a western philosophical background where medical ethics is mostly based on the rather fixed principlism framework (for a detailed analysis of this, see the discussion section).

### Background information

An idea of the socio-cultural, historical, educational and economic context of this country is helpful as this may influence ethical concepts as a whole and therefore the perception and the handling of concrete ethical issues in the local setting [36–38].

Gabon is a natural resource rich former French colony, which reached independence in 1960. It is located on the west coast of Central Africa at the equator. It has an estimated population of 1.67 million (2014 est.) with 86% (2011 est.) living in urban areas. 55%-75% are of Christian faith, the rest being animist, Muslim (< 1%) and others. The literacy level is estimated at 92.3% for males and 85.6% for females (1995). The official national language is French. It is taught in schools and spoken by the vast majority of the population. However the Gabonese people is composed of over 40 different Bantu tribes with partly completely different tongues. The infant mortality rate is 47 deaths/1000 live births and the life expectancy at birth is 52.06 years (2014 est.). The official HIV/AIDS adult prevalence rate is 4% (2012 est.) making Gabon the country with the 16th highest prevalence worldwide. The total expenditure on health is around 3.2% of Gabon’s GDP (2011). The physician density is 0.29 physicians/1000 (2004) compared to 3.5 physicians/1000 (2008) in Germany or 2.7 physicians/1000 (2004) in the USA. With a per capita GDP of $19,200 (2013 est.) “Gabon enjoys a per capita income four times that of most sub-Saharan African nations”. The unemployment rate is estimated at 21% (2006 est.) (all of the above information from [39]). Gabon’s Human Development Index (HDI) is 0.674 placing it well above the Sub-Saharan African average of 0.502 [40].

#### Financial situation of health care in Gabon

Unfortunately there is no official data available about the financial situation of the mentioned hospitals and the health insurance status in Gabon. According to study participants all three hospitals suffer from underfinancing and scarcity at different levels. Even though there are private health insurance plans and government health insurance programs like the “Caisse Nationale de Sécurité Sociale” (CNSS) interviewees pointed out that the vast majority of people is uninsured and has to pay cash to receive medical treatment. People who can afford it may go to private clinics or the military hospital outside of Libreville, which are far more expensive than public hospitals. The level of medical treatment was said to be highly dependent on the financial resources of the patient and his relatives or close peers (see Results section).

#### Traditional medicine

Traditional medicine has a strong background in Gabon and still plays an important role today [41]. Traditional healers can be found in rural areas as well as in the capital. Consequences of this like supernatural claims, non-evidence-based treatments, dosage inaccuracies and false diagnoses are common according to interviewees and the literature [13].

## Materials and Methods

Hospitals included in this research project were 1) the Centre Hospitalier de Libreville (CHL), the biggest and fairly well-equipped medical institution in Gabon serving Libreville’s 619,000 [39] inhabitants, and patients from the rest of the country, 2) the Hôpital Albert Schweitzer (HAS), a regional hospital located in the up-country city of Lambaréné with limited technical equipment and 3) the Hôpital Psychiatrique de Melen (HPM) just east of Libreville, which is the only public psychiatric hospital in Gabon. These health care institutions are a good representation of how health care is delivered to most of the people in Gabon. For example, people from all over the country often take great efforts when severely sick to get treated in the best-equipped public hospital, the CHL, as they hope to get better treatment there according to interviewees. Furthermore, we conducted interviews also at a rural hospital and at the only hospital for persons with mental illnesses in the country. The private health care sector isn’t accessible to a majority of the people because of high prices and wasn’t included in this study.

### Ethics Statement

We obtained the consent and approval of each of the three hospital administrations for our study, and to conduct the interviews on site. According to German and Gabonese regulations (Pharmaceutical and Medical Devices Laws, Medical Professional Law) no ethics approval is necessary for socio-empirical research that does not involve patients. The local IRBs at the three studied hospital sites in Gabon were involved in the process of developing our research project. Two members of the author group (GM and DSt) were involved in capacity building efforts for future members of the local IRBs. Prior to each interview the participants received an information sheet explaining the study rationale, the study design and the later reporting of anonymized data. The potential interviewees were then asked whether they were willing to participate in this interview research project. During the introduction phase of the interviews we explained again the above mentioned study rationale and the planned reporting of anonymized data and made sure that there were no misunderstandings. Consent was then considered to be implied when participants agreed to be interviewed and participated in the study.

Purposive sampling was employed to recruit participants covering different health care professions and clinical specialties. We interviewed 18 experienced medical professionals working in the following areas: internal medicine, infectious diseases including HIV/AIDS, intensive care, neonatology, pediatrics, gynecology and obstetrics, psychiatry, general outpatient clinics, social assistance and nursing management. The interviews were then held on site at these hospitals.

We used a semi-structured interview guide that included open questions about the understanding of clinical ethics, the ethical issues participants encounter in clinical practice, the practical relevance of these issues, and how they deal with them. A shortened English version of the interview guide can be found as supporting information (S2 File). The principal objective was to stimulate detailed reports on the general understanding of medical ethics, different experiences of clinical ethical issues and to learn what the participants found to be important without giving to much of a directive during the interviews.

Overall, 18 semi-structured in-depth interviews were conducted (n = 13 by DSi alone, n = 2 by DSi and DSt together, n = 2 by DSt and GM, n = 1 by DSt alone). Authors DSi and DSt had working experiences in hospitals in Gabon or another Sub-Saharan African country through clinical electives (3–10 months). The main data collection took place in November 2009, while a meeting to discuss results of the interview data with interviewees and other Gabonese health care professionals was held in November 2010. Eight interviews were held at the CHL, eight at the HAS and two at the HPM. The participants included ten physicians, six head nurses (including two midwives), one social worker, and one nurse manager (overall 11 females and seven males). All were experienced specialists in their respective fields who had been active for at least 3 years. Fourteen participants were of African and four of non-African (European and South American) origin.

The interviews were conducted and audio recorded in French in a face-to-face setting in the participants’ working environment. The duration of the interviews was between 19 and 67 minutes, with a mean of 37 minutes. 14 interviews lasted more that 32 minutes. Due to time restrictions of the participants 3 interviews were shorter than 32 minutes, with the shortest being 19 minutes long. Native French speakers transcribed the audio recordings, which resulted in an overall text volume of 72,135 words. The text analysis and coding was done in French. The entire code system and selected quotes used for this publication and its supporting information were then translated into English. MAXQDA (Version 10, VERBI GmbH) was used for qualitative data analysis.

A Grounded Theory (GT) [42] approach was used for the qualitative extraction of themes. At first, 12 interviews were read and studied intensively. Statements and text fragments were labeled and categorized into broad themes (open coding) and memos were added. In a second step, these interviews and their codings were read again, the main categories were connected through conceptual links, diversified and complemented by first and second order subcategories (axial coding). During this step the main categorical framework was developed. Afterwards, the remaining six interviews were analyzed using the same methods. No new main categories and first-order subcategories needed to be established; only second and third-order subcategories were amended. Therefore, category saturation and theoretical saturation were achieved for the main categories and first-order subcategories. This can be interpreted as evidence for the validity of the main categorical framework and a sample size of sufficient extent [42]. Because we were primarily interested in assessing the spectrum of clinical ethical issues in the studied care environments, we refrained from the third step of GT analysis (theoretical coding), in which the data are merged into more abstract theoretical categories and the emerging theory is refined.

The whole categorical framework was derived directly from the text using systematic text analysis and interpretation of statements and text fragments from the interviews.

After main data collection and analysis of the data we scheduled a meeting with participants of our interview study and other health care professionals and representatives of Gabon as well as local ethics scholars. This included the head of the national college of physicians (Ordre des Médecins du Gabon). The goal was to present our data, engage in a discussion about our findings with the above-mentioned participants, as well as clarify potential misunderstandings and misinterpretation of the data. After presenting our findings and discussing it with the 14 participants of the meeting no revisions to our core framework had to be made. We were assured of the face validity of our findings regarding the saturation of the spectrum of ethical issues experienced by health care professionals in the public health care sector as a whole.

## Results

**Fig 1:**
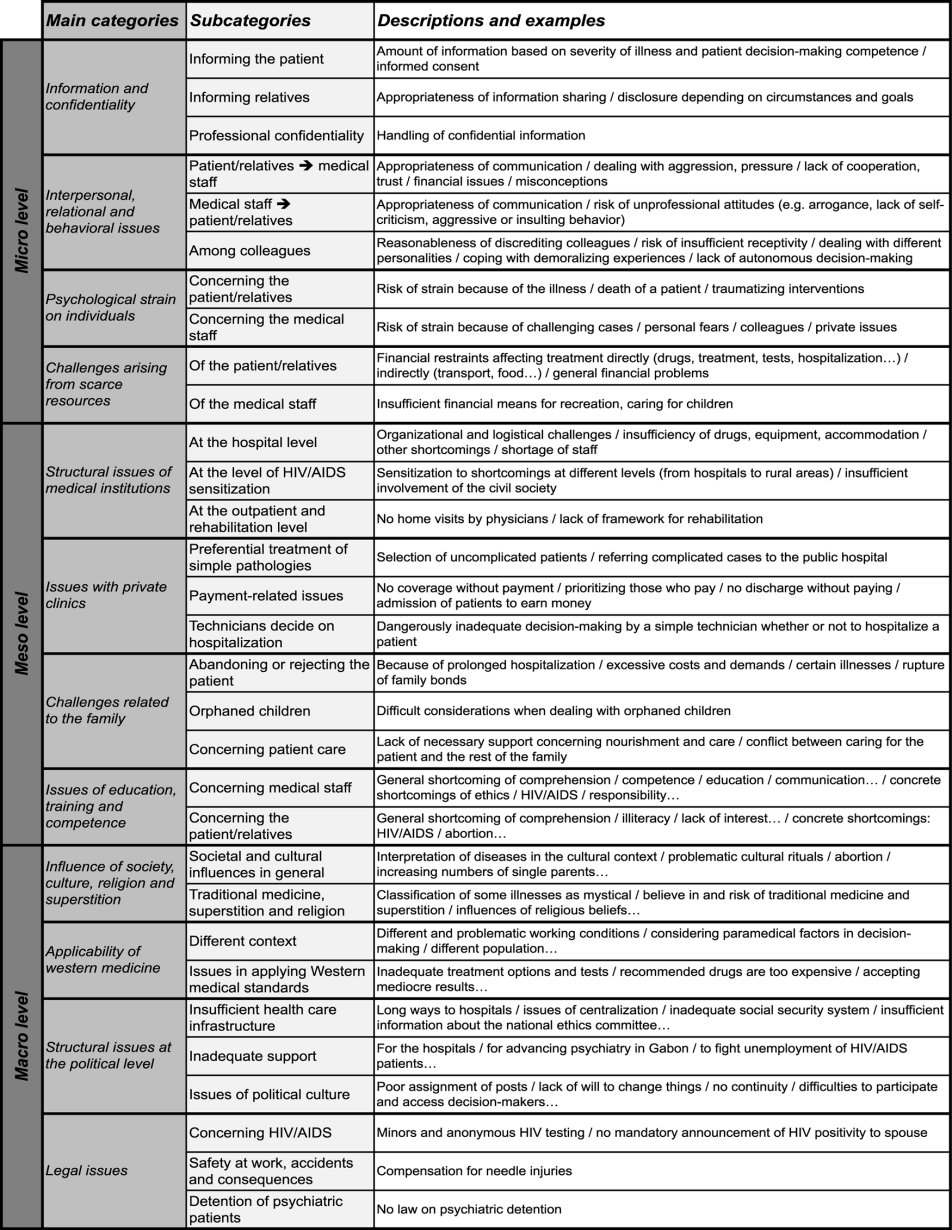
The spectrum of clinical ethical issues. Issues are grouped into three core categories, 12 main categories as well as 28 further-specified subcategories. Descriptions and examples of these subcategories are given. Issues are grouped to the level where they primarily arise.

Twelve main categories emerged describing issues that the interviewed health care professionals of Gabon encountered in their practice and which they classified as ethical issues. These could be grouped into three core categories according to where the issues primarily arise: the micro, meso and macro level of health care. Each of the 12 main categories consisted of two or three further-specified subcategories (28 first order subcategories in total). See Fig 1.

The micro level of health care comprises issues originating in the doctor—patient relationship, the interactions of clinical staff and the self-understanding of these individuals, as well as scarce resources of the patient, family and close friends. The meso level comprises issues with roots at the organizational and institutional level, for example hospitals, schools, education and training of health care personnel, as well as the family as institution. The macro level comprises issues arising in the cultural, sociological, religious, economic and political context.

All three levels are interdependent and therefore influence each other. For example, the issue of who is informed about an illness was described as being influenced by cultural and societal variables (macro level). But it is also related to where and how staff members were educated regarding professional communication and confidentiality (meso level). Finally, the physician decides at the micro level (in a specific working environment) how to handle patient information and confidentiality in relation to a specific patient.

For multiple reasons, it is impossible to display all the original quotes used to build the database underlying the categorical framework presented in Fig 1. For technical and didactic reasons we also refrain from presenting a more fine-grained framework with subcategories of second or third order as this would render the figure less comprehensible. However, a supporting information document containing a more comprehensive overview of the results and example quotes substantiating the categories can be found online (S1 File). In the following, we present some clinical ethical issues in more detail. We refrained from attributing the quotes to specific participant roles (nurse vs. physician, which hospital etc.) because this would harm our obligation to preserve participant anonymity as for example for some specialties there is only one physician at the HAS.

### Clinical ethical issues at the micro level

#### Confidentiality and disclosure of information

Several factors seem to influence the degree to which medical professionals informed the patient and their relatives. After analysis of the data we could recognize factors like the intellectual level, gender and psychological state of the patient and relatives, the medical condition and prognosis, and who paid for the treatment. We learned that the person who paid for the treatment often received more thorough information, sometimes even more than the patient him or herself, and at times without the patient’s consent. Concerning confidentiality in the context of HIV/AIDS, we received different answers ranging from the staff being *“obliged to tell the husband”* (if the partner was HIV positive), to being *“under oath”* thus keeping confidentiality, or a *“duty to persuade”* the patient to tell his or her partner.

#### Interpersonal, relational and behavioral issues

Several compliance issues were described as causing tension between the staff and patients/relatives, including leaving the hospital against medical advice, drug non-compliance, missing appointments, accusations and misbehavior arising from specific types of undesired diagnoses. Some participants found it difficult to accept that some patients were not interested in the disease and that some relatives focused mainly on the costs of care.

Several interviewees highlighted the risk of subordinating patient care to non-medical, secondary interests, or applying private convictions at work (e.g. regarding abortion). The quality of communication between staff and patients and their relatives was also criticized. Some physicians were said to work at private clinics besides working at the public hospital. One participant complained that physicians had their *“business”* running alongside. For her it was necessary to *“change the mentalities”* regarding working morality to improve patient care.

#### Psychological strain of individuals

We found out that psychological strain not only resulted from issues of the medical milieu in general (e.g. severe diagnosis, end of life decisions), but also from influences such as the lack of resources and mismanagement (see respective passages).

#### Scarce resources

Interviewees reported that because of financial constraints on the patient’s/relatives’ side, physicians sometimes could only *“do the minimum”* as there were no means to buy drugs, perform medical tests, or to hospitalize a patient with a severe illness. At times families were said to abandon patients because of high costs, which resulted in considerable psychological strain for everybody involved. While some saw the underlying problem in the lack of health insurance, social security (see macro) and general poverty, others thought that patients and their relatives also had a duty to contribute. Interestingly the interviewed staff didn’t directly complain about their own financial situations.

### Clinical ethical issues at the meso level

#### Structural issues of medical institutions

According to participants clinical ethical issues were often related to a general shortage of staff, especially of specialists (psychiatry, oncology, psychology, social workers). We were told that relatives had to take charge of nursing, nutrition and hygiene to complete the care package, which resulted in disadvantages for abandoned patients or patients without a large caring family. Furthermore, favoritism was described as playing a role in staff recruitment as well as in whether patients got admitted or not. All of the above were said to contribute to psychological strain on staff as well as on the patients and relatives.

Other ethical issues at the meso level included unequal access to health care, especially in rural areas, for example HIV/AIDS information programs, outpatient and rehabilitative care. Again, we learned that not all access to care issues were due to scarce resources. A major problem was seen in the inadequate management of the institutions’ available resources. Corruption and organizational incompetence resulted in inefficient utilization of already scarce resources according to participants: *“the technical level is probably not very developed*, *but with the minimum that we have*, *we could do a lot of things*.*”*

#### Issues with private clinics

While we didn’t interview the staff of private clinics, interviewees nonetheless related some ethical issues to the role of private clinics. It was argued that private clinics focused rather on *“simple pathologies”* and making money, sending away more complicated patients with more expensive and complicated conditions. These patients then had to be treated in a public hospital.

#### Issues related to the family

We considered patients’ families as social institutions and therefore placed these issues at the meso level. Supportive relatives were reported to be very important in case of serious illnesses, and high expectations were placed on the family, who often had to put aside other duties or other family members at home to care for the ill. We learned that this demand could *“paralyze”* the whole family. The interviewed health care professionals described their difficulties engaging in these complex interrelations and accepting families abandoning patients due to scarce resources: *“it’s true that people say the African family is big*, *but as soon as you are sick for more than a week at the hospital*, *you might have no more family”*. Besides the duration and cost, other reasons for abandonment of patients, that were mentioned, included the particular illness, especially in the case of HIV/AIDS and psychiatric illnesses. Further, medical professionals described moral distress when caring for orphaned children, as they often didn’t get any official support despite the goodwill of health care professionals and institutions.

#### Issues of education, training, competence and skills of staff

Interviewees regarded education, training, competence and skills as key elements for good health care and adequate conduct in ethically challenging cases. A participant highlighted an often-experienced lack of self-criticism by some staff members. She said that senior physicians mostly trained in France during the 1960s–1980s when physicians had a rather paternalistic and self-confident attitude towards their patients. After their specialization they would come back to Gabon, practice and teach their students how they had learned. Most participants said that they had not received any explicit ethics education, either at medical school or from continuing education. One participant argued: *“Ethics is only perceived as a research term*, *that’s all they know*, *but before arriving at research*, *there is ethics of everyday life”*. However all participants said that ethics and ethics education were important and should be improved.

*Issues of education*, *training*, *competence and skills of patients/relatives*: Education and competence were also considered to be important on the patients’ and relatives’ side. Several participants told us about manifold issues in this area. Uneducated patients and relatives also were said to cause conflicts as the staff sometimes had to “*submit”* to the lesser educated.

### Clinical ethical issues at the macro level

#### Influence of society, culture, religion and superstition

Interviewees reported that traditionally the family plays a central role in Gabonese society, which influenced how they informed the relatives, as they thought the family had a right to know what was going on. However, it was also described that this has been changing as the development into a more individualistic society could be seen. Interviewed health care practitioners faced dilemmas in situations where it was unclear to them whether to inform patients and relatives in the traditional way or not.

Old convictions still seemed to play an important role. One participant even said that they were *“imprisoned in the tradition”* as to how illnesses were perceived and treated. HIV/AIDS, cancer and mental disorders were still commonly seen as *“mystical illnesses”* by patients but also by health workers, as even some staff members described psychiatric illnesses as *“mystical”*. *“Mystical illnesses”* appeared as a punishment for wrongdoing to some. One example that was given is that one contracts HIV and its secondary diseases *“because you did something wrong* [in life]*”*. In consequence we learned that patients with *“mystical illnesses”*, as well as women who undergo illegal and disrespected abortions, risked stigmatization, abandonment and that these patients would often have a hard time to reintegrate into society once they recovered. These “mystical” beliefs must be seen as part of the local context and tradition. In many African traditional religions the concepts of spirits, ancestral spirits and spirit possession play an important role and are still very present today [43]. Sometimes these belief systems lead to clinical ethical issues as in the case of psychiatric illnesses that are often regarded as “mystical”. Health care professionals face ethical dilemmas in how to respond to these convictions.

One participant proposed that clinical ethics was a problem of *“rich countries”* and that there were more important issues to deal with before considering ethics more explicitly in medical training and continuing education. An example of a more pressing issue by this participant was the bad condition of medical equipment and the low level of advanced medical technology in Gabon. However several participants strongly disagreed and saw it as a very important topic that needed to be addressed in parallel.

### Applicability of western medicine

A physician suggested that western diagnostics and therapy guidelines weren’t applicable in Gabon because of a different population (majority of people with African origin vs. mostly Caucasian subjects in most clinical trials) and also a lack of resources (officially recommended treatment options often not available) leaving her without evidence-based treatment options. Because of resource limitations she said that she needed to pursue a *“mass policy”* for her patients rather than an *“individual policy”*, not doing justice to every patient.

### Structural issues at the political level

The health care institutions were described as being *“politicized”*, meaning highly influenced by politics. Interviewees saw this in a lack of continuity, with administrators who said to often be swapped for political reasons. Health care professionals at lower organizational levels reported it to be difficult to *“change”* anything.

#### Legal issues

We heard about a *“judicial vacuum”* that led to medical ethical issues. One example given was the handling of needle stick injuries and the lack of compensation for work injuries in general. Also the unclear judicial situation over the detention of psychiatric patients was seen as an issue, as it was reportedly relatively easy to lock someone up without legal restrictions, which gave a lot of power to psychiatrists.

## Discussion and Conclusion

This qualitative analysis of 18 in-depth interviews presents the qualitative spectrum of clinical ethical issues currently encountered by physicians, nurses, midwives, and social workers in different public health care environments in Gabon.

In most physician codices ethical issues refer to the ethical theory of principlism [44,45]. In the original theory for medical ethics principlism is based on the four basic principles of non-maleficence, beneficence, respect for autonomy, and justice. In the ethical theory of principlism these basic principles represent prima facie binding moral norms that one needs to obey unless they conflict with an equal or greater obligation in a particular case. When this approach is applied, the principles have to be specified and balanced against one another if a conflict arises. With respect to the principlism approach, an ethical issue in clinical practice might arise (a) because of the inadequate consideration of one or more of these ethical principles (for example: insufficient consideration of patient preferences in health care decisions) or (b) because of conflicts between two or more of the mentioned basic ethical principles (for example: balancing the principles of patient autonomy, non-maleficence and justice in a case where a HIV positive patient won’t tell the spouse of the sero-positivity potentially harming the partner). See also Strech et al. 2013 [46]. While other theoretical approaches for describing ethical issues exist, this is beyond the scope of this article. For an overview of concepts such as casuistry, virtue ethics or care ethics see for example Sugarman and Sulmasy (2010) [47].

For the interview study, however we refrained from introducing a specific understanding of ethical issues. First, it is questionable whether, for example, the core principles as defined in the original version of principlism, are the same in Gabon. Second, we aimed to not bias the interviewees’ responses. If we would have applied some sort of directive or narrow definition of what an ethical issue is beforehand, we could have missed certain issues and would not have been able to account for the full spectrum of clinical ethical issues. Framing the notion of “ethical issue” more broadly in the medical sector has been suggested before [48]. In our analysis of the interview transcripts we therefore accepted all described issues as ethical issues when 1) they were considered an “ethical issue” by the participant and/or 2) they involved some sort of moral decision making, weighing the consequences on either the micro (“personal level”, e.g. doctor-patient relationship), the meso (“institutional level”, e.g. the hospital), or the macro (“overarching level”, e.g. state or society) level.

In the introduction we mentioned that only few papers on clinical ethical issues in Sub-Saharan Africa can be found in the literature to date. Most studies on ethics in medicine deal with biomedical research ethics, which is to be seen as a distinct entity because of the different setting (research setting vs. everyday practice in the hospital setting). An example to clarify this is informed consent. One interviewee said that at her department she was *“the only one to do it”*, “it” being written informed consent before a potentially dangerous medical procedure. In clinical trials a far higher rate of informed consent for the same medical procedure is expected, as there is surveillance by a research ethics committee. This was also mentioned by an interviewee as already quoted in the results section: *“Ethics is only perceived as a research term*, *that’s all they know*, *but before arriving at research*, *there is ethics of everyday life”*. This indicates a need for studies that explicitly focus on clinical medical ethics; looking at everyday practice in the hospital setting. Another example for this difference in research vs. clinical setting is ethical issues arising from scarce resources on the patient side. This results in completely different ethical issues in research ethics compared to everyday clinical ethics at the hospital outside of clinical trials. While the problem of “incentives” to participate in a clinical trial for disadvantaged populations because of hope to get better and free treatment has been debated profoundly e.g. [49–51], the consequences of scarce resources on the patient side and the resulting ethical issues outside of studies is a different story that we reported in the results section.

This study is to our knowledge the first systematic analysis of the range of clinical ethical issues in a Sub-Saharan country. We define the spectrum of clinical ethical issues as a structured, qualitative account of ethical issues in the context of the specific setting (health care at public hospitals in Gabon), divided into broader categories and narrower subcategories that are based on text examples from the interviews held with health care professionals working in this setting. The purpose of our study is purely descriptive (‘empirical’ in its literal meaning).

Our findings demonstrate that scarce financial and human resources cause and influence clinical ethical issues in different ways and on interdependent levels of the health care system. However, the findings also show that clinical ethical issues in Gabon—and most probably also in other developing countries—cannot be reduced to problems of limited (financial) resources alone. The specific cultural, societal, and religious contexts give rise to specific clinical ethical issues such as confidentiality issues, challenging roles of relatives in medical decision-making, and the appropriate attitudes of health care professionals towards their patients. A striking example given above is the understanding of confidentiality within the family context playing a more important role than in contemporary “western culture”. This leads to an understanding of confidentiality that makes it “normal” for at least some health care professionals to share information with close relatives even without the permission of the patient. While many of these clinical ethical issues are well known and broadly discussed in the international bioethics literature [44,52,53], it is still unclear to what degree existing ethics guidelines [54], clinical ethics consultation models [55] and medical professionalism frameworks [45] (mostly produced by health care and bioethics institutions in an Anglo-American and Western European environment) can be transferred to and successfully implemented in African countries that face similar but differently shaped and contextualized ethical issues. We found many clinical ethical issues (see Fig 1 and additional supporting information S1 File) that seemed particular to the specific Sub-Saharan African cultural background and the professional self-understanding of the medical staff working in this environment. Furthermore, the understanding and conviction of how far physician confidentiality and other elements of medical professionalism should reach clearly varied among the participants. At the time of the study there existed no official Gabonese code of medical ethics or professional ethics for health care professionals in Gabon that would give an official framework for this [56].

In summary, the spectrum of clinical ethical issues encountered by health care professionals in Gabon, the dependence of ethical issues on cultural and social as well as financial aspects, and the varying attitudes of health care professionals towards ethical issues and different understandings thereof call for a more explicit discussion of and further research into clinical ethical issues in low-income countries.

Further research is needed to clarify and quantify the context-specific need for capacity building of health care professionals in clinical ethics and ethics consultation. If further research in other Sub-Saharan countries confirms our findings and also demonstrates a substantial need for capacity building, it needs to be assessed how such a training could be promoted in theory and practice at the level of medical schools and in continuing education.

At present, many international organizations such as the WHO and UNESCO focus on financing and organizing capacity building activities in research ethics for developing countries. To our knowledge no such infrastructure and research agenda exist for clinical ethics in low-income countries, nor for capacity building in this field. There is further need for research on the need for context-sensitive training and consultation models in this field for countries in Sub-Saharan Africa and most probably also other developing countries.

### Limitations

As this is a qualitative study answering a “what” question (“What is the spectrum of clinical ethical issues in Gabon?”), no quantitative conclusions on “how many” or “how much” questions can be drawn about the discussed topics. This study was conducted only in the west central African country of Gabon and therefore can only show the spectrum of clinical ethical issues in this country. Even though we expect similar findings in surrounding Sub-Saharan countries, the transferability can be questioned. Only health care professionals from public-sector institutions were interviewed. We focused on trained health care professionals and therefore didn’t include “traditional healers” who, especially in rural areas, are still frequented by locals. This exploratory study focused on attitudes and experiences from health care practitioners who work in the hospital setting. Patients and their relatives did not participate in the study; nor did politicians with responsibilities for health care. One of the authors (DSi) spent 10 months in Gabon at different hospitals (2 months at the CHL, 2 months at the HAS, and 1 month at the HIA-OBO (military hospital)) as a medical intern and he also visited other regional hospitals (Gamba and Franceville), spoke with health care professionals at these settings and therefore has gained further insides into how public health care is delivered in Gabon before the study was conducted. Furthermore one of the authors (ENA), who helped to analyze the findings, is a physician who was born in Cameroun where he also lived and finished his studies in medicine until the age of 28. The authors spoke with several health care professionals from Gabon to identify a purpose sample for this study (see Material and Method section). However there might still be an authors’ bias concerning the analysis of interviews and development of categories for the final spectrum of clinical ethical issues. A meeting with different health care professionals (including interview participants) was held 1 year after the main interviews to minimize this effect (see Materials and Methods).

## Supporting Information

S1 File Extended Categories and Quotes. Detailed list containing extended categories as well as more and longer quotes from the interviews.
(PDF)

S2 File Interview Guideline. A translated outline of the interview guideline used in the study.
(PDF)
