# Supplementary material for: Clinical Ethics in Gabon: The Spectrum of Clinical Ethical Issues Based on Findings from In-Depth Interviews at Three Public Hospitals
Source: PLoS One. 2015 Jul 10;10(7):e0132374. doi: 10.1371/journal.pone.0132374 (PMC4498757; doi:10.1371/journal.pone.0132374)
Supplement: S1 File — Detailed list containing extended categories as well as more and longer quotes from the interviews. (PDF) [file pone.0132374.s001.pdf]

Micro level

Information and confidentiality

Informing the patient

Degree of information depending on a certain medical condition or prognosis:
Newly discovered HIV/AIDS: *"you have to tell them"* | *"we don't hide it"*
After a stroke: *"the patient has to be informed"*
In case of cancer: *"we would not tell him, sir, you've got cancer"*
Other fatal diagnosis: *"We can't tell the patient that he's going to die, we won't say that."* | Some *"relativize"* the Diagnosis.
Reasons and explanations: *"still want to give the patient a joy of living"* | *"until the last day you have to encourage the patient"* | *"But if it's a cancer with a good prognosis, we explain to him, but not saying that's cancer. We tell him: We've discovered a lesion for which we have to give you chemotherapy ... to the family we say: It's cancer. We've told him that it's a lesion ... and when he gradually gets back to strength after the chemotherapy and ... we're satisfied, I tell him: you escaped; you're on remission of a cancer. He says: oh really, why didn't you tell me in the beginning? I say: if I had told you, you wouldn't have fought in the beginning."* | *"Do you have to openly tell the patient that he's going to die in two months or soon for example? You always have to say: it's going to be ok, it's going to be ok!"*
Depending on the patient as a person and on individual characteristics:
Psychological condition: No concrete information if the patient is *"psychologically weak"*
Gender: Rather dealing with male relatives than with female patients because: *"She can scream, will cry, throw herself on the floor, that's a little bothersome."*
Intellectual level: *"That depends on the intellectual level. You discuss with the patient and when you see he doesn't understand anything, it's not worth confusing him."* | *"... If the patient is intelligent, understands his illness and appreciates the situation, we talk."* | Contrary opinion: *"You have to discuss with the patient, you have to explain what you're doing, give him information, educate him."*
View of a participant of European background: *"The people here don't have the habit to explain a lot to the patient: What they are doing as treatment ... or intervention ... they don't always give explanations ... they don't verify if the patient has well understood."*
Written informed consent:
Not very common: A physician reported that in her department she was *"the only one to do it [written informed consent]"*.

Informing relatives

Disclosure depending on circumstances and goals:
Intellectual level: *"We will not tell the relatives if they don't understand."*
Closeness: Some only inform the *"closest relatives"*
Responsibility: Some only inform *"the most responsible [person]"*
Financially responsible: *"the person that pays"*
With or without knowledge of the patient: *"Generally we ask the patient if he wants us to tell to his family about his worries ... but sometimes we don't even ask about the patient's opinion, especially in the case when the patient doesn't take care of himself [financially] and a relative pays."* | While not always telling the patient the truth, some *"always talk to the relatives"*
Informing the spouse about HIV/AIDS: *"We are obliged to tell the man that his girlfriend or his wife: Has she told you that she is seropositive?"* vs. *"If we ... know a seropositive wife, we don't want to tell her husband if the wife hasn't spoken out about it. We are under oath"*, but there is a *"duty to persuade"* the patient to tell the spouse.

Professional confidentiality

Handling of confidential information:
Concerning patients/relatives: *"They [the relatives] don't have that concept of medical confidentiality anymore which we have a duty to keep ... really, this is a big problem."*
Concerning the staff: *"there are some who lose the concept of medical confidentiality"*

Especially with HIV/AIDS: *"Those, who come to do a test so that they know their latest serological status, often don't come back to get their results because there is no medical confidentiality at the healthcare institutions."*

Interpersonal, relational and behavioral issues

Patient/relatives → medical staff

Compliance: *"they can come to one appointment, the next time they don't come"* | *"some patients don't take their medication regularly"* | *"they want to do traditional treatments and continue the modern treatment"*
Behavioral issues concerning HIV/AIDS: *"there are even those who don't want for example ... that their wife is up to date ... that he is HIV infected."*
Financial issues: some *"refuse to pay"* after receiving hospital services | relatives sometimes aren't *"interested in the pathology"* as they focus on the *"the financial side of things"*
Aggression/pressure/communication: "*They aggress the midwife"* after the death of a newborn |*"threatening"*, *"insulting"* and *"pressuring"* of staff members | *"There are a lot of problems on the communication level between persons, meaning the people here – that's my opinion actually – have problems in communicating with each other and in explaining things. They are immediately in an aggressive mood."*

Medical staff → patient/relatives

Risk of unprofessional attitude (e.g. arrogance, lack of self-criticism, aggressive or insulting behavior): *"They [the staff] are not responsible enough"* | *"Often the nursing care isn't done"* | *"When it's after 10 o'clock in the morning and you send a person to the laboratory, nobody will do your analysis ... they say: Come tomorrow morning because we have already finished. And they know that ... there are a lot of patients."* | *"There is also a lot of value judgment concerning the women who do that [abortion], they aren't well considered at the maternity ward ... a woman who did a self-induced abortion, is not always well received because she is judged according to this and they don't try to understand why..."* | *"And that's where ethics begin, to have a critical mind: Is that, what I'm doing, good? If it's good, is it good for the physician or is it good for the patient?"* | *"Our patients complain sometimes about our behavior towards them, when you arrive at the hospital, a lot of times, there are some that act a little like a boss ... he [the medical professional] has all the power, so the patient is not to ask anything, the patient doesn't have any right at the hospital."* | *"But the medical culture, it comes through compassion, to put yourself at the service of others. And not to say: hey, I have an appointment; I have my business that's waiting! No, we need to change the mentalities."*
Appropriateness of communication: *"People aren't very, let's say diplomatic in how they say things, they are pretty direct"* | *"There is the issue of communication. How to inform the relatives, how to take them, how to announce ..."* | *"Aggressive"* behavior and *"insult of the patient/the relatives"*

Among colleagues

Dealing with different personalities and coping with demoralizing experiences: Reports of *"difficulties bound to interpersonal relations between the colleagues who don't see things the same way"*, which also *"demoralizes you a little because you are trying to have a certain attitude and you don't understand why the others aren't like you"*. | Physicians with *"strong personalities, who without discussion have the tendency to impose their point of view"* | Being seen *"as a danger"* because you *"brought in new methods that didn't fit into their view"*
Risk of insufficient receptivity: *"Little receptivity of physicians"*

Psychological strain on individuals

Concerning the patient/relatives

Risk of strain because of the illness: Difficulties to *"accept"* the diagnosed illness or its consequences, e.g. *"it's not easy that firstly the patient himself accepts that we remove his leg or his arm"* | *"Acceptance of the status"* (HIV positive)
Incurable diseases: *"When the patient doesn't recover, this also discourages the relatives."*

Concerning the medical staff

Personal fears: *"Fear to call into question nursing practices"* | fear that *"they would be contaminated"* when in contact with HIV/AIDS patients
Risk of strain because of challenging cases: Death of patients: *"it pains you, morally you're hurt"* | *"psychologically everybody at his level has his burden to carry"*
Being *"confronted with people who live with suffering and poverty"* or who *"have lost hope"* | *"Hard to tell"* the relatives when the patient died
Influence of private issues: *"Sometimes you're sad ... it's hard to do your work ... sometimes you might be aggressive because you're tired, you have so many personal worries ... I'm coming to work with a set of problems that in the end may have the effect that my work won't be done well altogether."*

Challenges arising from scarce resources

Of the patient/relatives

Financial restraints affecting treatment directly (drugs, treatment, tests, hospitalization…): / indirectly (transport, food…) / general financial problems: *"Seeing the financial constrains – that's often the case – a lot of times we are endowed to do the minimum. Well, at the limit you have the impression, that when somebody comes to the hospital, and it's not malaria or tuberculosis, it gets complicated."* | *"So that's right, we are led to choose the treatments, to choose the additional medical tests on basis of the financial resources of the people…"* | *"if there is no help [from the hospital], the patient dies"* (because he can't pay for the treatment) | *"So it happens often that we have patients who have pretty serious pathologies, but who can't support themselves because there's no more money. It's not that we can't treat them but rather that there's no money. At this stage, well, we're blocked; we can't go forward, so we tell the people that they have to go home because there's no more money. That's very difficult as a situation."* | *"Well, seeing that he didn't have any money, we couldn't propose him any treatment and we said he needs to go home."* | *"We have diagnosed the cancer but we don't have the means to treat it. We'll take him to Libreville knowing that this person doesn't have the means to get himself treated because it's very expensive to get yourself treated against cancer in Gabon."* | *"There are other departments of the hospital doing exactions. It's written on all the walls: no receipt, no right to enter, no hospitalization or no surgical intervention."* | *"Now, the hospital: it takes too long, it's a lot of money, I think it's the same thing, they will breakup the plaster, lift up the patient and say that they will do that at the indigene."* | *"So he left the ward, he was well balanced [regarding the glycaemia] and then afterwards it was trashy. So it's the follow-up of the patients when they leave the hospital which is more complicated [because of multiple reasons later given in the conversation e.g. no money to come regularly to the hospital]"* | *"Otherwise it's the patient's problem, because he has got to have the money so that he can go to Libreville."*
Consequences of a strike at the CHL: *"The center for hemodialysis in Libreville closed its doors. There are people who died of renal failure because they didn't have enough money to get treated in private clinics. This, these are things that are completely inconceivable for me."*
View that patients can't expect "free" treatment: *"Yes, in fact, it has to be, the patient or the relatives of the patient also have to participate in "health". We too, we participate and we also give something from our collaboration ... the patients are sometimes impoverished ... that's really hard."*
'Non-medical' consequences of scarce resources on the patient's / relatives' side: *"Sometimes you also have stressed relatives always facing the lack of financial means who* [the relatives] *can then be aggressive…"* | *"Here in Africa we say: hungry bellies have no ears. So you will talk to someone who takes his medication who knows his serological status [HIV positive], but if he doesn't know how to eat, when he doesn't know*

where to sleep, I don't think that he will understand you, he will nod his head to say that he has understood, but he hasn't understood anything." | *"Well, the problem that we face often, is, that sometimes when we disclose the illness of the patient a little, sometimes the relatives, they can leave despite everything and say: in this case, I don't take charge, that's a little too much, that's more that I can afford, I don't continue. So this is bad for the patient and then he might run away and that's complicated."* | *"This* [patients who don't pay] *causes a big shortage of earnings for the institution because ¾ of the patients don't manage to pay."*

Of the medical staff

Lack of financial means for childcare: *"it's too expensive"*
Lack of financial means for adequate leisure activities and recreation: *"And as the people don't have enough money to pay for leisure activities, which are extremely expensive, the people only do "work – home". At what moment does this break apart?"*

Meso level

Structural issues of medical institutions

At the hospital level

Insufficiency of and problems with drugs, tests and equipment: *"I would insist on this – this being poverty –, frankly, this is a big problem because it can happen that on the ward or at the hospital level, we lack pharmaceutical products and the patient, too, he doesn't have the financial means to buy at the pharmacy. These are especially the problems we encounter regularly on the ward."* | *"These are concerns that we could manage relatively easy, if we had a hospital that is sufficiently equipped."* | complaints about a general *"lack of equipment"* or *"adequate equipment to treat the patient"*: *"we continue to take blood with a simple needle ... you know the risk of needle injuries that the medical staff takes in the face of HIV patients?"* | Equipment that *"breaks down all the time"*, *"displays wrong results"* | lack of *"maintenance"* of equipment | *"We don't mange to resuscitate a patient because of scarce resources. It's blocked like in the village actually. We get by with what we have. And then, we have more deaths than people who survive. We register a lot of deaths."* | General *"shortage of drugs"* | Problems to *"make the correct and definitive diagnosis"* because of a lack of additional tests. | Misunderstandings with the relatives because of lack of means: *"The relatives might think that we neglected him, while it's a lack of means [on the hospital side] to save the patient."*

Insufficiencies regarding accommodation and room: Sometimes *"even placed the children on the floor"* | *"It's hard for the doctor at the emergency room of the pediatric department to tell this women who has a baby that is convulsing in the arms: Madame, come back tomorrow, there's no room today. And altogether knowing that this woman who came to the hospital, if you send her away, she couldn't go anywhere else. Since she doesn't have money to go somewhere else."* | Sometimes they *"don't have a choice"* when there is no *"vacant bed"* | Premature discharge because of lack of room: *"Normally you have to keep the patients for one month, but we keep them for 10 days because there's a lack of room."* | Poor condition of rooms: *"The principal ethical problem that I have is the unhealthiness of our rooms on the ward. I very, very, very rarely hospitalize. I'm one of the oldest physicians of the department and it's me who hospitalizes the least because I wouldn't like to sleep in these rooms here."*

Psychological strain for the staff because of insufficiencies on the hospital side: *"Morally, psychologically, you, the staff that works, you are affected. Basically you have no choice ... but it hurts. It gets to you, but you can't do anything facing this situation."* | *"It hurts you, when you couldn't have the right product that might have saved the patient ... really that makes you cry."*

Lack of staff: *"We're in need of medical personnel. We're broadly understaffed."* | *"Only two nurses for 32 hospitalized patients and at the same time the emergency cases... hence, this is a difficulty, it's very very tough."* | especially regarding some specialties: *"Until there is evidence to the contrary: We have only a single woman among Gabonese physicians, who is a psychiatrist, only a single Gabonese ... a single psychiatric physician for Gabon, really, that's insignificant!"* | *"We don't have a psychologist, we don't have anybody from the social service. All this is lacking."* | *"We have always talked about a psychologist here at the hospital and who finally doesn't exist. Because we have very serious cases, where we are in need of a psychologist who, for example / the cases of AIDS patients: You necessarily need a psychologist who has to discuss at length, who has to have time..."*

Overwork of staff and its consequences: *"Sometimes, it's a little bit the overwork that makes that the staff can be a little aggressive."* | *"We have a lot of work, so it's true you always have to give yourself the time to reflect, well, we don't always have the time, so everyone thinks in his corner."*

Organizational and logistical challenges / other shortcomings: Lack of support for poor patients from the hospital: *"if there is no help [from the hospital], the patient dies"* | no complete care coverage: *"What we are doing, is the medical care, but it's not exhaustive for a patient because there are a lot of shortfalls. But the care, it's not only the medical prescription. There has to be hygiene, care for hygiene, there has to be nourishment which can accompany the medical prescription."* | Continuing education: *"We don't have teaching because we don't have sufficient means"* | Young physicians who work at the hospital sometimes *"don't yet have a salary"*

Poor resource utilization: *"It's true, we're in an African country where the technical level is probably not very developed, but with the minimum that we have, we could do a lot of things."* | *"A simple question of organization. We could already advance. Anyway, we have to get better ... for example try to improve our profitability level in the department.* | Issues of non-competence driven staff recruitment: *"For long time we*

have done recruitments based on affinity, because you're the cousin of, the son of etc. and from another side and not necessarily because you're care giver ... so we don't see that much competence – in my opinion – at the level of recruitment."
Issues because of bad organization of payment process at the hospital: *"There is often the attempt to directly take money from the relatives. You know that's an ethical problem and one of morals because it's practically theft ... this means when a patient arrives there are the once that are called to ask for money, meaning: you pay me so I can get you through for this or that ... this is a problem you have to fight!"* | *"The people, when they suffer, when they arrive at the hospital, they're in hope already and when we ask them to deal with, all the administrative hassles actually, that's tough on the morale ... this can be delicate psychologically."* | *"So they accepted to take my sister [who didn't fulfill the admission criteria] because it was my sister, and I, I worked at the hospital. [If it was] someone else? They would have said: No, wait at the admission office, you need the papers!"* | Issues when treating patients for free: *"Sometimes it's difficult for us because we depend on an administration that takes money from our salary to pay the patients' fees. And we sometimes / we would really like to treat but we don't have the right…"*

At the level of HIV/AIDS sensitization
Sensitization shortcomings at the hospitals: *"as we are not a sensitization service, in the end we can only do what we can, here it's the medical work"*
Sensitization shortcomings in rural areas: *"Did they go to all the corner of Gabon…? Gabon is not only Libreville…"*
Insufficient involvement of the civil society: *"My observation is that the health institution, which means, the medical staff thinks that HIV/AIDS is their business because they are physicians, they are nurses, they are midwifes, they have diplomas from "grandes écoles" [elite schools]. So they think that it's not necessary to implicate the civil society. So they sometimes want to do everything. But today we have the prevalence, which is very high, which today is at 8% and we really have the obligation to implicate the whole civil society, to implicate the players, to implicate the people living with HIV in order put the fight in the center, to control the prevalence in Gabon. So I've noticed myself ... that there is still a lot to do with the health institutions because there is no close collaboration with the association, the civil society and the health institutions. There is something like a war, actually like a lack of trust."*

At the outpatient and rehabilitation level
No home visits by physicians: *"But unfortunately we can't do home visits ... I would really like this because it's this that allows us to really see if it's getting better or not ... There are a lot of lost cases of patients [patients that don't show up at the hospital again] even with hepatitis, even with tuberculosis, even with hypertension, even with HIV ... there are a lot of lost cases ... you can't do all the work together, it's sad."*
Lack of framework for rehabilitation: *"it is hard to do things regarding rehabilitation"*.

Issues with private clinics

Preferential treatment of simple pathologies
Selection of uncomplicated patients / referring complicated cases to the public hospital:
"So what I noticed, my little experience: These are people for relatively simple pathologies, but when it gets complicated as when they [patients] *get renal or cardiac failure, generally, as soon as there are problems, they end up here at the general hospital. So when you have money, you go to a private clinic, it's ok when it's for one week or two weeks, but when it lasts, they end up here at the hospital and we can only cry."*

Payment-related issues
No coverage without payment / prioritizing those who pay: *"At the other institutions here in Libreville, even before they ask: Why are you coming to the hospital? They ask you: do you have money? That is the question that they ask you at the [private] hospitals and that's difficult."* | They *"refuse to take the patient because he doesn't have money and they ask for money first."* | *"They will take the one who can pay ... if there are other children with the same worries, they will treat the one who can pay, for the other they will say: We're sorry!"*

No discharge without paying: *"You have been healed, stabilized but they don't let you leave as long as you haven't paid ... I don't think that's good. It's not good because the people, who stay on the ward, don't just occupy a bed, but it also increases the bill and if he couldn't pay for 8 days, it's not like he will pay a hundred days. So you lose and you penalize the other patients."*
Only taking patients to earn money: *"it is for a story of money"*

Technicians decide on hospitalization
Dangerous inadequate decision-making by a simple technician whether or not to hospitalize a patient: *"When you arrive at a private clinic…you don't even have access to the physician, you have access to the reception and at the reception it's an ordinary employee. These are not necessarily people from the medical body."*

Challenges related to the family

Abandoning or rejecting the patient
Rupture of family bonds: *"I see old persons, the family is there, children, small children, everybody comes. Even if we say that he will die, they will accompany him to the last day. And others, well, they come, they drop [the patient] off…you tell yourself it's the poverty that does this. It's the breaking of family connections, if the people don't love each other…otherwise you have the people who are surrounded and this problem doesn't occur."*
Because of prolonged hospitalization: *"But when he [the patient] is all alone, it's difficult because not only you are in pain, but you have to pay, thus you have to have a good family. And well, it's true that people say the African family is big, but as soon as you are sick for more than a week at the hospital, you have no more family. Generally it's your father/your mother, who remains close, the others are gone. Oh yes, this is how it goes here!"*
Certain illnesses: HIV/AIDS: *"there is always the problem of rejection of this patient"* | Psychiatric illnesses: *"When they are tired of managing their patients in their neighborhood, they come, they bring them here to the hospital, they abandon them, they don't come back to visit them, they don't come back at all, even when the patient is stabilized, they don't come to get them."* | Issues of reintegration after treatment of psychiatric illnesses: *"We have always difficulties to reinsert them into their family, since the families dissociate themselves."*

Orphaned children
Difficult considerations when dealing with orphan children: *"It's difficult, because additionally he is an orphan, it's that he isn't capable of deciding, thus if his parents aren't here either – I haven't said that you have to take a decision for him – but you have to take a decision ... I think in this area we can't be systematic, well, disease, test for everybody, treatment for everybody ... I haven't solved this problem, I'm still in the midst of an ethical reflection about it."*

Concerning patient care
Lack of necessary support concerning nourishment and care: Patients have to be *"assisted"* and *"accompanied"* which sometimes isn't done.
Conflict of caring for the patient or the rest of the family: *"Others evoke that they have left other children at home and they can't continue for a long time because there's the one who is sick who has to be saved and those who are healthy: What do you have to choose? But well, they make a decision like this and then they leave."* | *"When a person in a family suffers from Buruli, it's almost the whole family that is paralyzed because the mother can't work anymore, the father can't really make an effort…it's difficult. Sometimes he works in Libreville, the others are in Lambaréné, he has to come all the time."*
Issues to find blood donors for the patient among family members: *"To already find two donors around of your family… they have an apprehension to come and donate blood to others ... they are afraid: Not me, not me…"*

Issues of education, training and competence

Concerning medical staff

General shortcoming of comprehension/competence/education/training: *"the important issues, they lie in the training of the staff"* | The need for adequate equipment and competent staff go *"hand in hand"* | *"Because even if I have all the [material] means to treat the patients well, but if I don't have competent staff members who understand what they are doing, what they have to do, I risk / it's a catastrophe."* | *"At the staff level not only the effective number, but also the competence, the effectiveness of this staff regarding the patient."* | *"Well, ethical challenges, as I said, it depends on the training. Thus, if the staff is trained better, if the staff members understand what we expect from them, yes, we have already solved a problem. The staff, when I speak about the staff, I mean everybody."*

Issues of main medical education: Teachers *"weren't effective"*, didn't *"teach well"*, reports of *"frightening teacher"* who *"insulted"* students and were *"aggressive"* | *"…lack of personnel, teachers, really qualified ones"* | *"Yes, because during training, they have to teach the individuals exactly what we are: we are called upon to help others, to be acquainted with them, to understand them and to accept them, how they are at the moment and exactly to help them get out."*

Issues of continuing education: *"So, seeing that there is no system of continuing education today, the people read what they want. Hence someone may have a subscription for a medical journal, but there are some that do this and others who don't."* | *"We have very little retraining, training, go and see somewhere else how things have evolved. This is another domain, continuing education, it would be desirable at the same time for the physicians and especially for the paramedical staff."* | *"A nurse who has left school, 10, 20 years ago, who is in the field should benefit from an internal seminary to put her/him back to the level, to renews the knowledge."* | *"When the physician go to seminaries, the nurses also need to go, we all should be educated because in the field we are collaborators. It shouldn't happen that the nurse is always lost facing what the physician does."*

Issues of training in communication: *"The problem arises at the level / maybe the communication problem at the level of the staff, to better communicate with the relatives because these are people who are already anxious regarding their patient. But everything comes from / there has to be training / the problem of the communication of the staff."* | *"If I would have had for example a training, maybe I would have better found the words."*

Lack of self-critical thinking: *"I think, I'm the only one trained according to the North American school and all my colleagues are trained according to the European school and especially the French school. According to the French school the physician comes after God the father, what the physician decided can't be questioned by anybody. The patient has to accept and take everything for granted what the physician said. The patient must not question what the physician said."* | *"…they haven't been educated in this sense, to questions themselves"* | Reports of *"little receptivity of physicians"* for new ideas and methods.

Education and information of the patient: *"It's not that they are evil or bad. They haven't taught them that you should do it like this and especially their mentor. And this is because it works like this: according to the European school, you have a mentor to think, then you copy that what the mentor does. And they haven't seen their mentor do this. Thus for them, what they are doing is good since they redo what their mentor does. That means they will neither doubt nor have a critical mind, even regarding what they are doing."*

Separating private and work issues: *"A nursing staff that doesn't know that family problems stay at home and work problems are problems of work / private problems are private problems and this man or woman who doesn't know how to differentiate this conduct in this way, is always frustrating the patient…I think there's a lot to do, there's a lot to revise with the nursing staff, to be able to lead them, to reeducate them so that they understand that there are home / family problems* [and] *problems of work…"*

Lack of knowledge about HIV/AIDS: *"I would take the case of people living with HIV/AIDS, these people, when they arrive to renew their medication, they have always been frustrated, be it because the nurse doesn't have enough knowledge or the midwife doesn't have enough knowledge in the field of AIDS and because of this ignorance she dares to frustrate the patient or say to the patient: Anyway, it's not my fault that you have AIDS."*

Lack of medical ethics education: *"Unfortunately there are not enough people informed about medical ethics."* | *"When we studied this course [medical ethics] didn't exist…it's only now that it's been introduced, so a lot of us don't yet really correctly perceive all that is to put under the topic 'medical ethics'."* | some think that ethics committees *"create a blockage…because they don't know what ethics are"* | *"Hence, here, ethics is only perceived as a research term, that's all they know, but before arriving at research, there is*

ethics of everyday life." | "There should be an ethics education and this should also be reinforced because…as ethics is well anchored the physician knows where he is heading, because if the physician doesn't know the problem and this comes back to his responsibility, you might be mislead." | "I think that the people [the staff] aren't necessarily up to date on patient rights, human rights etc. even the charter of values, and I think that the people have an intellectual background that doesn't allow them to understand or they haven't gotten the occasion to be educated to understand certain things." | "I think that there is a restraint referring to fear. Already the people may not have well understood what ethics is, too. What does this mean? And then for me this especially is – I think – to call into question. To say at a certain time: where am I? Is this good?"

Concerning the patient / relatives

General shortcoming of comprehension and education: *"there are shortcomings at the level of comprehension between us and the patients" | "The people [patients and relatives], they don't always understand" | "You also need to have people in front of you who understand you" | "We often have to submit a little to the difficulty because – you know – it's an issue of education. Each person comes with his education, each persons understands, there are those who don't understand." | "But this culture [good medical culture], it should be anchored not only at the level of the physicians, but at the level of the medical staff and at the level of the patients, too. They should learn that they have rights and duties. Therefore this is a whole culture, a whole mentality to develop, to change and I think it's possible."*

Lack of interest in the pathology: *"The people don't think in terms of pathology…the people haven't yet integrated this aspect of things…I ask the parents to read…You have to inform yourself a little…This aspect isn't yet fixed in the minds: The illness at the start can influence a whole life!"*

Lack of understanding creating situations of strain for the staff: *"This is a job that we do with love…certainly, if a baby leaves [parents taking the baby home] like this, you're hurt, you even want to quit because you know in your head that this child will die, that this child will die under terrible conditions…certainly this depresses you a little, to see parents who don't understand."*

Illiteracy: *"The people don't know how to read here, are you going to ask them for a written informed consent? Tell them what you would like to do. But the given word is important."*

HIV/AIDS: Some still *"refuse to believe that AIDS exists" | "For example in the village, the people don't know. They have heard of AIDS, but they don't know the ways for contamination, what you have to do, if you need to do a voluntary screening." | "The moms who have been tested seropositive can't understand their seropositivity, so that they don't do the follow up correctly as they should." | "Sometimes it's difficult, when a mom can't understand the benefit based on the implementation of a treatment. Thus this gets complicated for the nursing staff because you don't know to which saint to vow and how to make this patient understand so that she can accept the legitimacy of things." | "You can say that some moms can understand, but others don't understand…sometimes I'm very annoyed by the moms, when I notice that a mom has abandoned her treatment."*

Macro level

Influence of society, culture, religion and superstition

Societal and cultural influences in general

Interpretation of diseases in the cultural context and consequences for everyday practice: *"culture obliges you to observe a certain number of rituals…if you haven't watched these rites you can run into trouble in the days to come"* | There are *"certain customs"* to follow. | *"But the western world becomes more and more individualistic meaning that the human being is singled out even if you announce an illness you have to ask for the patient's permission if another person can be informed of that matter. But we, in Africa particularly, according to our traditional principal…a human being is always a child…because one is a descendent of one's father and mother…so when somebody is ill, we don't think that he or she is the one who is responsible. We don't have to address him or her but the family, father and mother…it's them that have to see and participate in decisions."* | *"In the western world, it's the individual that counts, meaning that you only tell the individual, not to the family. If there is a death, the father can say: I wasn't up to date. I didn't know that he had such a problem. Thus you can't announce the death to the maternal family because the father is the boss, it's him who has to take up the responsibilities."*

Reasons for issues with HIV/AIDS: *"sex is taboo"* in Gabon | *"A mother can't have the courage to talk to her son openly how you get infected by AIDS"* as she would be *"ashamed"* | *"At the moment AIDS is a disease that is taboo in our tradition"* | Often men have *"an official wife"* but *"several girlfriends besides"* | *"The majority of towns is sensitized in Gabon, but we have a stubborn people in front of us, a group that refuses completely to believe that AIDS is an illness like all others"*; some people *"don't see AIDS as an illness."*

Reasons for issues with mental illnesses: *"The big problem that often arises in our hospitals is the division of things, that you have to make of what is truly medical and what you can put on the account of culture…we're often confronted with this dichotomy…the patient, even some of us, some of the staff, are convinced that this, what happened, that such an illness is only to put on the account of culture…then it's sometimes hard to convince the patient and sometimes even the staff members…that this comes from the psychiatric pathology and that is purely cultural."* | *"We all have somewhat been raised in this culture…even having all the scientific things preconscious, it's sometimes difficult to make the distinction between what is simply cultural and that what is really medical…here at the hospital we talk science, meaning that what we have to convey, what we have to tell the patient, the relatives, has to relate to what we have studied."* | *"Most people consider the psychiatric hospital like an asylum. This means, they come abandon [a patient] and disappear…they go home, they don't come back to get their patient."* |*"When someone suffers from a mental illness, he is no more useful for the society…so the people have always problems to be rehabilitated."* | *"People think that when someone has behavioral problems, well, he's done, he can't resume work. But we have people here who we accompanied, stabilized…the people don't understand exactly the importance of the psychiatric hospital, the importance of mental illnesses."*

Different concept of pain as part of the disease and treatment: pain isn't only *"part of the illness"*, but also *"part of the treatment"*. | *"You find this in Buddhism as well: when you're in pain, that's because you have done something bad, thus you 'pay' a little necessarily of what you did. But here you heal you have to be in pain."* | Consequences are *"big problems to establish a preventive analgesic treatment."* | *"During childbirth you never do a peridural [epidural anesthesia] here for the delivery. This is inconceivable because you have to give birth in pain, it's part of it."* | *"Thus at the level of pain management there's enormous work to do. It's at the cultural level, at the religious level, at the level of beliefs…"*

More and more single parents without support: "*many single parent families"* | *"It is difficult these are young women, who live alone, who have children, who fight… for many things"* | *"The teenagers are a little bit left alone pregnant"* | *"But we nevertheless see young mothers abandoning their children…they give birth, they abandon and bam, they leave…they run away. Or well, there are those who we picked up at the last moment: She has left her baby and she even left to the city. This is a phenomenon that becomes recurrent…these are the things we see now because of the phenomena that I have cited you."*

Difficulties with abortion (which is officially illegal in Gabon): *"The women aren't well-thought-of when they do this sort of things…and then afterwards when they try to have children, they have difficulties. This, too, a woman without children isn't well-thought-of either."* | *"There will be a lot to do in regard to ethics,*

in regard to women rights, in regard to all that is [family] planning, the right to abortion, contraception, because this a country where there is no family planning…there is also a lot of value judgment regarding the women who do this."

Traditional medicine, superstition and religion

Classification of some illnesses as mystical: *"we're in a black country where there is a lot of witchcraft meaning where people believe in witchcraft."* | *"We in Africa, we have a problem with witchcraft."* | *"Here in Gabon, we are still imprisoned in the tradition because you have a fever, you can't get up and immediately go to the hospital, no. At first you will collect the leaves and do warm baths, scrub the body. In medicine even a higher temperature, a child will convulse, when you convulse, the brain takes a hit, you can even go into a coma. But no, you don't see it like this. But no, we are really rooted in our tradition. This is the problem in Gabon, [only] a handful of people will say: Yes, the hospital."*
Issues with traditional medicine: *"dosages aren't controlled"* which may result in *"intoxications"* | *"The big problem that arises is mostly at the moment when the child is not well, the parents, they try to do other things and this creates always incomprehension because they sometimes want to continue our treatment and then take the traditional treatment."* | *"When the disease has evolved and there are complications"*, e.g. a child is in a critical state *"the parents might think: it's no more the dictum of modern medicine, we have to take the child elsewhere, to the Nganga [traditional healer]."* | *You can't lie to yourself, there are people that think too much of the 'Nganga'[traditional healer], hence of the fetishist, of the charlatan."* | *"They go there [to a traditional healer]. You don't find healthiness, it's death that follows and the relatives remain in regret."* | *"We know what's going on, there are lots of people – you have to do statistics – we know, many people have lost their lives because they went to traditional healers. People have fixed ideas, they say that these are the vampires who eat them, thus it's there [at the traditional healer] where they can find life."*
Even some physicians believe in traditional medicine: In the case of *"a disease that hasn't been cured at the hospital…[and] the physician has tried to do the best, [and] it hasn't worked"* some might say: *"Try go to an indigenous healer!"*
HIV/AIDS as a mystical illness: *"As you talked about cancer. All this goes into the same direction: Always mysticism. For example we here concerning AIDS, we get Kaposi's sarcoma, we get lymphomas. These are the types of cancer we get here. You see someone who comes with a big limb with Kaposi papules and even when he comes here, the people will tell you that it's the 'fusil nocturne'* a mystical idea that afflicts you in the night, because you did something wrong | *"They won't say that it's the AIDS virus that is the cause of all this."* | *"A lot of patient don't accept it [HIV/AIDS] as an illness like all others. It's a mystical illness!"* | *"A father, aged sixty or older will say: But it has been more that 10 years that I haven't had any sexual intercourse…he won't understand. It's mystical."* | *"The traditional healer can't say that it's AIDS. He will say that it's the 'fusil nocturne'* [see explanation above] *or something someone brought upon you…if you have problems in your family, he will jump onto that. He will say that it's your uncle, it's your father, it's also your colleague who has brought this upon you."*
Mental illnesses as mystical illnesses: *"A patient arrives; he says that he hears voices, that he sees bizarre things. Well, you will be told that this aren't hallucinations, these are rather bad spirits that attack him."* | *"First of all, in Africa the mental illness is considered an African illness. What does this mean? This means that when someone manifests behavioral troubles, firstly you come back to the traditional healer."*

Applicability of western medicine

Different context

Different and problematic working conditions / Considering paramedical factors in decision-making: *"What we have in Africa isn't the same thing that you have in Europe, but some years ago, in the 1950s until the 1980s – which isn't long ago – there were somewhat the same worries in Europe compared to what we have in Africa today."* | You have *"choices to make"* and *"include other human factors"* you don't include *"in Europe"* you're *"nevertheless in Africa with particular working condition in a particular milieu"*.
Different population: *"We have specific populations, OK? The clinical trails are done – you have to know them – on Caucasian populations and now you try to include women, but essentially on men. OK, we have an Afro-African population and we have a lot of women in our populations because the men don't live that long in Africa … thus you have to well integrate this."*

Issues in applying Western medical standards

Inadequate treatment options and tests/recommended drugs are too expensive: *"So your guidelines, I like it when you [western physicians] do your conferences, but when you're here at the CHL, the public hospital, where the people have an average of 200 000 Franc CFA [305 €, $ 387], forget this!"* | *"The big problem I have here, are the American heart guidelines…what they have said, here, the newest molecule that you should give, but how much does that cost? Who are you treating here? You're treating yourself, your physician's conscience: because I have followed the guidelines! But you don't treat the patient, who won't buy it [the medication]."*

Surrounding factors that make it hard to practice like in the Western World: *"All this is due to issues of logistics. This is the big problem and it's this that leads to ethical problems because we're often confronted if someone can pay or not, if someone is surrounded or not, the doctor will act according to this. So we are very dependent on surrounding factors of the patient here. It's not that we have just the patient or the disease."* Consequence: You're satisfied with a *"good average"* or *"fairly well"* result, you do *"more of a mass policy than an individual policy"*.

Structural issues at the political level

Insufficient health care infrastructure

Long ways to hospitals, too centralized, which influence treatment decisions: if the patient lives *"in the bush, very far away"* | some patients don't want to get hospitalized because *"they live far away"* | *"At the moment, the psychiatric hospital of Libreville [Melen], is the only health care institution that treats mentally ill patients in Gabon, this is a difficult problem…generally the patient shouldn't be far away from his family"*, resulting in issues when trying to *"put the patient back into his family environment"*

Long work ways for the staff and lack of good public transportation: "*sometimes [it's] a little difficult"* to get to work, also *"causing delays"*.

Issues with the HIV/AIDS sensitization program: lack of *"means of transportation"*, field workers *"can't arrive in the last corners"* of the country. | *"You have to decentralize this management, you have to make the associations autonomous, you have to allow that the civil society gets involved concerning the mobilization for AIDS prevention in Gabon."*

Lack of service to bring in psychiatric patients: Psychiatry staff *"has to play two roles…the people who have to get them by force and play at the same time psychiatrist or care giver at the hospital"* creating issues of trust regarding the patients.

Insufficient information about the national ethics committee: *"I think they have established a Gabonese ethics committee last year. I don't know."* | *"I only know that there is the ethics committee, but what have they published?"*

Inadequate social security system: *"The main difficulty is to take care of patients properly since there is no social security system as in a lot of European countries. When the patients come here, they have to pay for themselves. So they not only pay for the hospitalization, accommodation, room, but they have to pay for everything like medication, everything that we request at the hospital, additional tests, ... they have to pay for it."* | *"Listen to the politics: We will create an emerging country, we want to go ahead. But you can't do anything in a country where there are not sufficient human resources . The people who are sick have to get cared for correctly. They can't go to work ... so I think you have to start somewhere and above all you have to start with the health care institutions, before taking care of everything else, improve the health care for individuals ... to have strong and 'valid' persons who can work to bring the country forward. Otherwise we won't make progress."* | *"One of the main consequences is the mortality that remains very significant. We are limited by miscellaneous factors…for the adults here, there is a diminution of yield because someone who is sick and comes to the hospital and we couldn't care for properly, even if his condition stabilizes after some time of hospitalization…we might ask him to go home, this person won't be able to work anymore…this person will only be a burden for the society, he won't be able to contribute anymore. Thus this is the vicious circle. If you want to develop a country, you have to 'develop' the health of the people."*

Inadequate support

Supporting the hospitals: *"the ministry should help us…the health minister should make an effort to help the hospitals"* | During a strike politicians called to reopen the CHL: *"You have to open the wards! How do you*

want to open a ward if there is no oxygen? How do you want to open an intensive care unit, if there are no monitors? How do you want to open an emergency room if you don't have the minimal equipment? This is an ethical problem because on the one side the politics forces you to open because it looks good to say 'it works', then on the other side you're tied up with that…because you can't hospitalize, because you hospitalized and then you can't administer the care necessary. This is a problem, a big ethical problem."
Advancing psychiatry in Gabon: *"The government doesn't support the introduction of the psychiatry in Gabon!"*
Inadequate help and issues of unemployment of HIV/AIDS patients: Lack of *"social aid, taking into account the daily life"*, rather helping HIV-positive patients *"to find work"* than *"putting a lot of money into campaigns"*

Issues of political culture

Poor assignment of posts: *"You have to assign the right person that is needed to the position: namely a physician, namely a person living with HIV, namely a psychologist, namely a social assistant…Thus you have to assign these men to their positions this way you can really help, bring what is needed where it is needed. But when you assign someone who has nothing to do with this, who has no competence in this and you give him responsibilities in health, he won't be able to bring what is needed where it is needed."* | *"Hence this is a whole culture, a whole mentality that has to evolve, to change and I think this is possible. But it's only possible with people who know where they are going, not with people that will be appointed by decree to an ethics committee and they have no idea of what ethics is."* | *"The people err through ignorance, they don't know, the people that are appointed, the decision makers err through ignorance. They aren't worse than others, they err through ignorance."*
Lack of continuity: projects with the family ministry but no knowledge *"if the project has come into being because afterwards they 'replaced' this woman"*
Issues with political institutions: They are *"politicized"* | Lack of interest to *"contribute something to change this"* | No *"free access"* to these institutions so that *"people could present their projects, to express themselves to receive aid or to make things move"*.

Legal issues

Concerning HIV/AIDS

No mandatory announcement of HIV positivity to spouse: *"the legislator"* needs to make a law that *"all partners announce"* their seropositivity to their respective partner, as a lot of *"those who are sick don't tell their partner"*.
Minors and anonymous HIV testing: *"What do you have to do? Take care of them or wait until they are accompanied by a parent?"*

Safety at work, accidents and consequences

Compensation after needle injuries: "Judicial vacuum" as there are no laws concerning this | *"We have people who are victims of needle stich injuries, that is to say nurses or physicians who have pricked themselves and there is no law that compensates them."*

Detention of psychiatric patients

No law how to handle psychiatric detention: Elsewhere it is strictly *"codified to take someone to the hospital with force"* and you have to *"obtain an authorization"* | The psychiatrist can easily *"lock up"* someone when *"dangerous for himself or for the society…namely when the patient [was] aggressive"*.
