# Supplementary material for: Clinical Ethics in Gabon: The Spectrum of Clinical Ethical Issues Based on Findings from In-Depth Interviews at Three Public Hospitals
Source: PLoS One. 2015 Jul 10;10(7):e0132374. doi: 10.1371/journal.pone.0132374 (PMC4498757; doi:10.1371/journal.pone.0132374)
Supplement: S2 File — A translated outline of the interview guideline used in the study. (PDF) [file pone.0132374.s002.pdf]

<u>Interview Guideline</u>, Sippel D, Marckmann G, Ndzie-Atangana E, Strech D

1 Introduction
- o Introduction of interviewer
- o Short presentation of the project
- o Explication of planed anonymized data usage

2 Duty, function and working area of interviewee

3 Ethical and general issues in every day practice
- o General challenges in every day practice
- o Understanding of "ethics" and "ethical issues"
- o Ethical challenges in every day practice
- o Ethical challenges in other areas of health care in Gabon

4 Judging/assessing ethical issues
- o How the beforehand described issues or challenges influence every day work
- o How ethical challenges are being dealt with
- o Difficulties in dealing with those challenges

5 Thoughts, suggestions, comments, wishes, fears
- o Concerning the project
- o Concerning ethical issues related to the current situation of health care in Gabon

6 Acknowledgement and farewell
